# Ogilvie syndrome: peculiar manifestation of acquired immunodeficiency syndrome in non-institutionalized middle age female in Tanzania

**DOI:** 10.11604/pamj.2020.37.298.25252

**Published:** 2020-12-02

**Authors:** Ahmed Mussa Jusabani, Mubashir Alavi Jusabani, Dhanji Karsan Patel, Dilipkumar Anant Pradhan, Kaushik Laxmidas Ramaiya, Salim Ramzan Surani

**Affiliations:** 1 Department of Radiology, The Aga Khan Hospital Dar es Salaam, Tanzania,; 2 Department of Orthopaedics, Kilimanjaro Christian Medical Centre, Kilimanjaro, Tanzania,; 3 Department of Surgery, Shree Hindu Mandal Hospital, Dar es Salaam, Tanzania,; 4 Department of Radiology, Shree Hindu Mandal Hospital, Dar es Salaam, Tanzania,; 5 Department of Medicine, Shree Hindu Mandal Hospital, Dar es Salaam, Tanzania,; 6 Internal Medicine, Corpus Christi Medical Center, Corpus Christi, United State of America and Internal Medicine, University of North Texas, Dallas, United State of America

**Keywords:** Ogilvie syndrome, acute colonic, pseudo-obstruction, acquired Immunodeficiency syndrome, case report

## Abstract

Since it was first documented in 1948 by Sir William Heneage Ogilvie, numerous cases of Ogilvie syndrome have been described in literature due to various medical and surgical causes. Nonetheless, only a handful of cases only have been documented due to underlying Acquired Immunodeficiency Syndrome (AIDS). A 41-year-old female was admitted with an acute abdomen secondary to partial mechanical intestinal obstruction or paralytic ileus based on signs and symptoms and Abdominal X-Ray (AXR). She was known to be HIV/AIDS WHO clinical stage II on treatment. On diagnostic imaging studies she had distended large bowels without features of mechanical intestinal obstruction and the diagnosis of Ogilvie syndrome was suspected after other differentials were excluded. Early recognition and appropriate management are essential, because if left untreated the bowel distension may progress to caecal perforation and fatal peritonitis. Medical imaging with Computer Tomography (CT) scan and colonoscopy has helped in achieving an accurate diagnosis and avoiding unnecessary laparotomies. Although an uncommon disorder, for earlier and accurate diagnosis a high index of suspicion is required by clinicians and radiologists who are treating patients with underlying HIV/AIDS. Ogilvie’s syndrome is a rare condition and if missed can be fatal. In patients with HIV/AIDS, the symptoms may be directly due to HIV infection, secondary to opportunistic infections or possible neurotoxic effects of HIV treatment or lack of vitamin and minerals. It is important to exclude Ogilvie syndrome in patients from surgical causes of the acute abdomen to avoid unnecessary surgical procedures.

## Introduction

Ogilvie's syndrome is a rare acquired disorder most commonly reported in patients in the sixth decade and is more common in men [1]. It occurs predominantly in patients who are hospitalized or institutionalized. The symptoms of Ogilvie syndrome mimic those of mechanical obstruction of the colon, but no such obstruction is present [2]. It is caused by an unclear disturbance to the autonomic innervation of the distal colon and is often associated with other different conditions [3]. Ogilvie's syndrome or acute colonic pseudo-obstruction is a motility disorder characterized by acute and progressive colonic distension. This syndrome usually occurs in hospitalized patients with underlying several medical or surgical diseases with unclear pathophysiology. Diagnosis is usually established by the clinical history, physical examination, and radiological findings on plain abdominal X-ray [4]. This is a rare condition and review of literature in PubMed and Google Scholar did not reveal any case of adult Ogilvie's syndrome documented from sub-Saharan Africa in association with under-lying HIV/AIDS. We hereby report a case of a 41-year-old female with this condition.

## Patient and observation

A 41-year-old female patient was admitted to our facility with a gradual worsening of colicky lower abdominal pains, distension, and constipation for five days. She was passing flatus and had no fever, nausea, or vomiting. She denied the use of any antibiotics in the last few weeks preceding her symptoms. She was a known patient with AIDS on nevirapine and zidovudine. She is married living with her husband and had two children. She was a housewife, non-smoker, and occasionally drank alcohol. She had no history of hypertension, diabetes, drug allergy, or any prior surgery. On admission, she was afebrile, pink conjunctiva, not jaundiced, or dehydrated and with no oral thrush. Abdominal examination revealed diffuse moderately distended abdomen with mild central tenderness but no rebound tenderness. There was no organomegaly or any palpable mass. The bowel sounds were slightly reduced. The digital rectal exam revealed hard stools in the rectum. A review of other systems was normal and there was no obvious skin rash noted.

**Figure 1:**
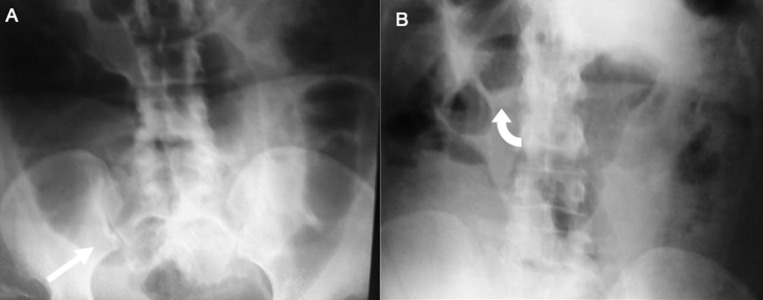
AXR supine (A) and erect (B) views showing dilated large intestine and in particular the caecum (asterix) with the normal small intestine caliber and no abnormal air-fluid pattern (curved arrow)

**Figure 2:**
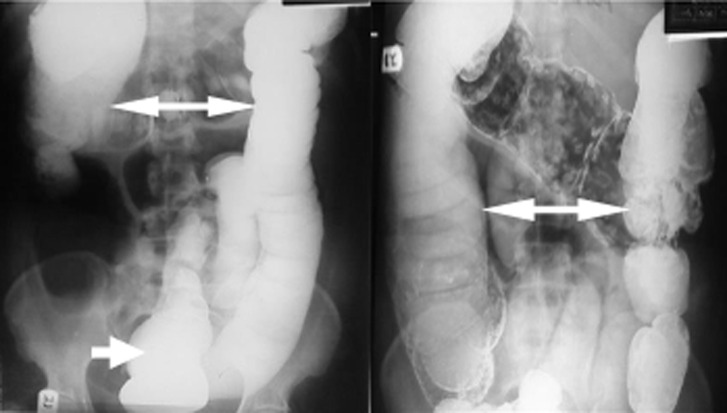
barium enema study showing dilated large intestine (bidirectional arrows) without evidence of mechanical obstruction. There is continuous filling down to the rectum (short arrow)

**Figure 3:**
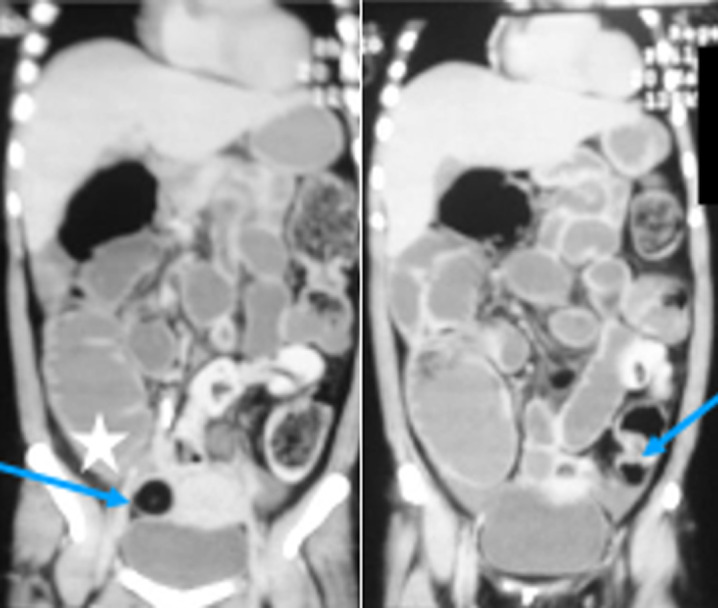
coronal views of CT scan reveal dilated large intestine up to caecum (asterix) with no zone of transition, normal small bowel caliber, and no ascites or pneumoperitoneum. Incidentally, the patient was incidentally found to have bilateral adnexal dermoid cysts (blue arrows)

**Figure 4:**
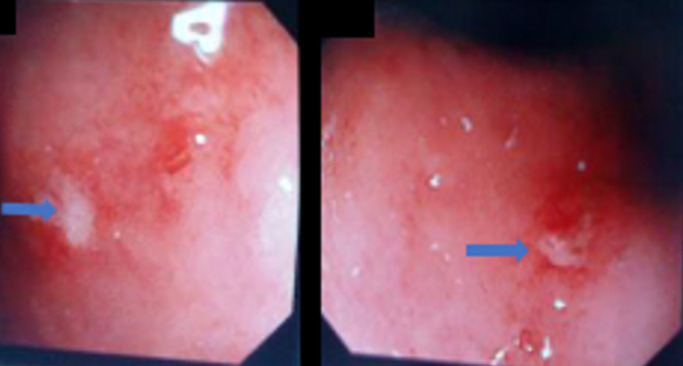
colonoscopy images revealing two superficial ulcers (arrowheads) in the rectum the histology of which revealed non-specific inflammatory changes

The initial working diagnosis was paralytic ileus due to unknown etiology. Laboratory investigations revealed a hemoglobin level of 11.8g/dl and white blood cell counts, both total and differential were within normal limits. Random blood sugar levels, electrolytes, lipid profile, liver and renal profiles, serum amylase as well as urinalysis were all within the normal range. AXR revealed dilated large bowel with normal small bowels and no abnormal air-fluid levels (Figure 1). Abdominal ultrasound revealed dilated bowels filled with fluid and had sluggish movements. Barium enema study demonstrated dilated large intestine without evidence of mechanical obstruction (Figure 2). CT scan was also performed and revealed dilated large intestine without any zone of transition and normal wall thickness, small bowels were normal and no ascites or pneumoperitoneum (Figure 3). The patient also underwent colonoscopy which could only be scoped up to 100cm due to poor bowel preparation. However, two superficial ulcers were seen in the rectum (Figure 4) and biopsy was obtained and the results revealed non-specific inflammatory changes. The patient was treated with intravenous fluids and kept nil per oral for five days and thereafter gradually started on oral feed as tolerated. Following the clinical improvement of symptoms, she was discharged and on subsequent outpatient follow up she did not report to have the same symptoms.

## Discussion

Ogilvie syndrome or Acute Colonic Pseudo-obstruction (ACPO) is a clinical disorder characterized by clinical features and radiographic appearance of proximal large bowel dilatation with marked abdominal distension but without evidence of distal colonic obstruction (Neuro-mechanical Dissociation). The colon may become massively dilated; if not decompressed, the patient risks bowel ischemia, perforation, peritonitis, and death [5-7]. In 1948, Ogilvie described two patients with metastatic cancer and retroperitoneal spread to the celiac plexus. The patients also had signs and symptoms of colonic obstruction but with no evidence of organic obstruction to the intestinal flow. Ogilvie hypothesized that the etiology of their conditions was an imbalance in the autonomic nervous system with sympathetic deprivation to the colon, leading to unopposed parasympathetic tone and regional contraction, with resulting functional obstruction [1]. This is a rare clinical condition that makes it difficult to gather data regarding its occurrence and there is no data available to suggest a different frequency according to race. Some researchers suggest that this illness may have a slight male predominance and it is a disease of elderly patients, usually older than sixty years; however, it can also occur in younger patients, particularly those with underlying severe medical or surgical disorders [2-3]. The disease is common in patients with a history of trauma, recent surgery, severe pulmonary disease, severe electrolyte disturbance, severe cardiovascular disorder, advanced malignancy, severe systemic infection and patients on medications such as narcotics, anticholinergics, amphetamines, phenothiazines and steroids [6]. Usually the patients with this condition present with abdominal pain (80%), nausea and vomiting (60%), Constipation (40%), and fever (37%). The majority (90-100%) will have abdominal distension and in about two thirds there will be non-specific abdominal tenderness. Bowel sounds may be normal or hyperactive in 40% and hypoactive, high pitched, or absent (60%) [7].

Generally, the overall medical status of patients with Ogilvie syndrome is poor. The prognosis in patients successfully treated for this disorder is directly related to the severity of the co-occurring medical conditions. The risk of perforation is higher with a larger caecal diameter (10-12cm). Mortality rates of 15% have been reported. However, it is high in the presence of perforation and peritonitis which increases the mortality rates to 40% [8, 9]. With increased awareness, better diagnostic tools, and prompt management of this disorder, mortality rates have decreased. The etiopathophysiology of this disease is not very clear with some theories suggestive of; autonomic denervation, splanchnic hypo-perfusion, metabolic dysmotility, pharmacological, hormonal, and infectious causes [10]. An imbalance in the autonomic innervation appears to lead to a functional bowel obstruction, as supported by pharmacologic and spinal blockade studies, metabolic abnormalities, and retroperitoneal trauma. Some current evidence suggests that an interruption of the sacral parasympathetic nerves leads to an adynamic distal colon that is similar to Hirschsprung´s disease, except with normal ganglion cells observable on autopsy [11]. Other research supports the belief that the sympathetic tone increases in patients, who are usually very ill, leading to inhibition of colonic motility [10]. The cecum is the usual site of the largest dilatation in patients with Ogilvie syndrome and, thus, is more prone to the risk of perforation. With its larger diameter, the caecum requires the smallest amount of pressure to increase in size and wall tension and thus perforation can occur. This is explained by Pascal´s Law and Laplace´s Law [12, 13]. Therefore, a dilated intestinal segment has a greater wall tension than a non-dilated segment; if the dilatation and tension are sufficiently great, blood flow may be obstructed and ischemia of the bowel will occur and resultant perforation and peritonitis.

**Medical imaging findings in patients with Ogilvie syndrome**: plain and upright abdominal X-rays are the most useful diagnostic tool for this disorder. AXR shows a dilated colon, often from the cecum to the splenic flexure, and occasionally to the rectum. Serial films may be used to follow the clinical course and the response to treatment. Specific attention given to the diameter of the colon is important. If the colonic diameter exceeds 10 cm, decompression of the colon must be considered and expedited. In addition to caecal diameter, the duration of distension also appears to be an important factor in perforation risk. Gastrografin enema: is water-soluble and has a high osmolarity with laxative properties; therefore, this contrast medium tends to cause a fluid shift into the colon and may subsequently increase colonic motility. A gastrografin enema may be both diagnostic and therapeutic for this disorder. Given the nature of pseudo-obstruction, air should not be instilled into the colon if the enema is performed. While a CT scan is not frequently required to establish a diagnosis, it may be helpful in the objective assessment of caecum excluding the presence of frank perforation, peritonitis, obstruction, and toxic megacolon. The hallmark of colonic pseudo-obstruction is the presence of dilatation of the large bowel (often marked) without evidence of an abrupt transition point or mechanically obstructing lesion. Colonoscopy may be helpful both diagnostically and therapeutically. This single procedure can help exclude an obstructive process and decompress the colon in approximately 80 percent of patients [14]. It may be technically difficult because of the difficulty in adequately preparing the colon to allow good endoscopic visualization.

**Association between Ogilvie´s syndrome and HIV/AIDS**: HIV/AIDS is a chronic debilitating condition that can lead to various multi-organ manifestations which can be of very serious consequences and such patients may be prone to develop Ogilvie syndrome. In patients with HIV/AIDS Autonomic nervous system effects can be potentially due to various reasons including infection with HIV or concomitant CMV which may lead to demyelination or axonal degeneration. Malnutrition from various nutritional deficiencies but specifically lack of vitamin B12 leads to neuropathy. The highly active antiretroviral therapeutic agents are known to be a neurotoxin and can produce neuropathies [15]. Several studies have reported an etiopathogenic association between Herpes Zoster and Ogilvie Syndrome in the absence or presence of HIV/AIDS due to the simultaneous occurrence of herpetiform rash and pseudo-obstruction as well as the rapid resolution of the abdominal dilation and the functional recovery from the colonic pseudo-obstruction after antiviral therapy. There also has been the demonstration of varicella-zoster virus infection by immunohistochemistry and viral particles in the muscularis propria and myenteric plexi of the colon in an HIV-positive patient with herpes zoster and Ogilvie's syndrome [16-20]. The incident patient was a middle-aged female who was well-nourished and not hospitalized or institutionalized without any acute severe medical or surgical illness. However, she presented with clinical and imaging features of Ogilvie syndrome and had underlying acquired immunodeficiency syndrome. In the absence of any other cause, HIV is assumed as a possible etiological factor.

## Conclusion

Ogilvie´s syndrome is a rare condition and if missed can be fatal. There has to be a high degree of clinical suspicion to diagnose early in patients with chronic medical illnesses. Diagnostic imaging plays an important role in the diagnosis. Decompression of bowels by flatus tubes or colonoscopy is important to avoid complications as perforation and peritonitis and hence decrease the mortality. In patients with HIV/AIDS, the symptoms may be directly due to HIV infection, secondary to opportunistic infections or possible neurotoxic effects of HIV treatment or lack of vitamin and minerals. It is important to exclude Ogilvie syndrome in patients from surgical causes of acute abdomen and avoid unnecessary surgical procedures.
